# Submerged cultivation and phytochemical analysis of medicinal mushrooms (*Trametes* sp.)

**DOI:** 10.3389/ffunb.2024.1414349

**Published:** 2024-06-11

**Authors:** Malay Srivastava, Moni Kumari, Santosh Kumar Karn, Anne Bhambri, Vivek G. Mahale, Sushma Mahale

**Affiliations:** ^1^M/s Xcel Life Sciences, Fremont, CA, United States; ^2^Department of Biochemistry and Biotechnology, Sardar Bhagwan Singh University, Dehradun, India; ^3^Department of Biotechnology, Shri Guru Ram Rai University, Dehradun, Uttarakhand, India

**Keywords:** *Trametes* sp., β-glucan, phytochemicals, flavonoid, phenol, saponin

## Abstract

Mushrooms are widely available around the world and have various nutritional as well as therapeutic values. Many Asian cultures believe that medicinal mushrooms can prolong life and improve vitality. This study aims to characterize the phytochemical and polysaccharide content, mainly β-glucan content, of mycelial biomass and fruiting bodies collected from the Himalayan region, particularly Uttarakhand. Through molecular analysis of the LSU F/R-rDNA fragment sequence and phylogenetic analysis, the strain was identified as *Trametes* sp. We performed screening of phytochemicals and polysaccharides in mushroom and biomass extracts using high-performance liquid chromatography (HPLC) and a PC-based UV-Vis spectrophotometer. The macrofungal biomass was found to be high in saponin, anthraquinone, total phenolic, flavonoid, and β-glucan content. In biomass extract, we observed a high level of saponin (70.6µg/mL), anthraquinone (14.5µg/mL), total phenolic (12.45 µg/mL), and flavonoid (9.500 µg/mL) content. Furthermore, we examined the contents of alkaloids, tannins, terpenoids, and sterols in the biomass and mushroom extracts; the concentration of these compounds in the ethanol extract tested was minimal. We also looked for antioxidant activity, which is determined in terms of the IC_50_ value. *Trametes* sp. mushroom extract exhibits higher DPPH radical scavenging activity (62.9% at 0.5 mg/mL) than biomass extract (59.19% at 0.5 mg/mL). We also analyzed β-glucan in *Trametes* sp. from both mushroom and biomass extracts. The biomass extract showed a higher β-glucan content of 1.713 mg/mL than the mushroom extract, which is 1.671 mg/mL. Furthermore, β-glucan analysis was confirmed by the Megazyme β-glucan assay kit from both biomass and mushroom extract of *Trametes* sp. β-glucans have a promising future in cancer treatment as adjuncts to conventional medicines. Producing pure β-glucans for the market is challenging because 90–95% of β glucan sold nowadays is thought to be manipulated or counterfeit. The present study supports the recommendation of *Trametes* sp. as rich in β-glucan, protein, phytochemicals, and antioxidant activities that help individuals with cancer, diabetes, obesity, etc.

## Graphical Abstract

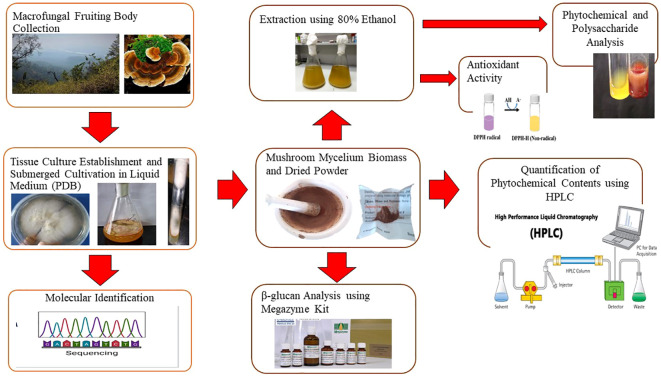

## Highlights

- Obtained a high quantity of *Trametes* sp. biomass in submerged culture.
- *Trametes* sp. showed a high β-glucan content in macrofungal biomass extract (42%).
- Tested *Trametes* sp. biomass and mushroom extracts were high in protein and phytochemicals such as saponins, anthraquinones, total phenolic content (TPC), and total flavonoid content (TFC), and low in fat content.
- The biomass and mushroom extracts also exhibit high antioxidant activity.

## 1 Introduction

In recent years, the higher fungi of the *Agaricomycetes* class (*Basidiomycota* division) have been found to have a wide range of natural, structurally diverse bioactive compounds with promising nutritional and therapeutic properties. These compounds have attracted the interest of researchers studying medicine worldwide because of their unique sensory qualities, health benefits, and pharmacological activity due to their synthesizing bioactive metabolites (Stastny et al., 2022). Basidiomycetes produce a variety of bioactive substances, such as lectins, phenolics, homo- and hetero-polysaccharides, flavonoids, terpenoids, sterols, and volatile organic compounds (Lin et al., 2019; Lu et al., 2020; Sevindik et al., 2020; Yadav and Negi, 2021).

**Figure 1:**
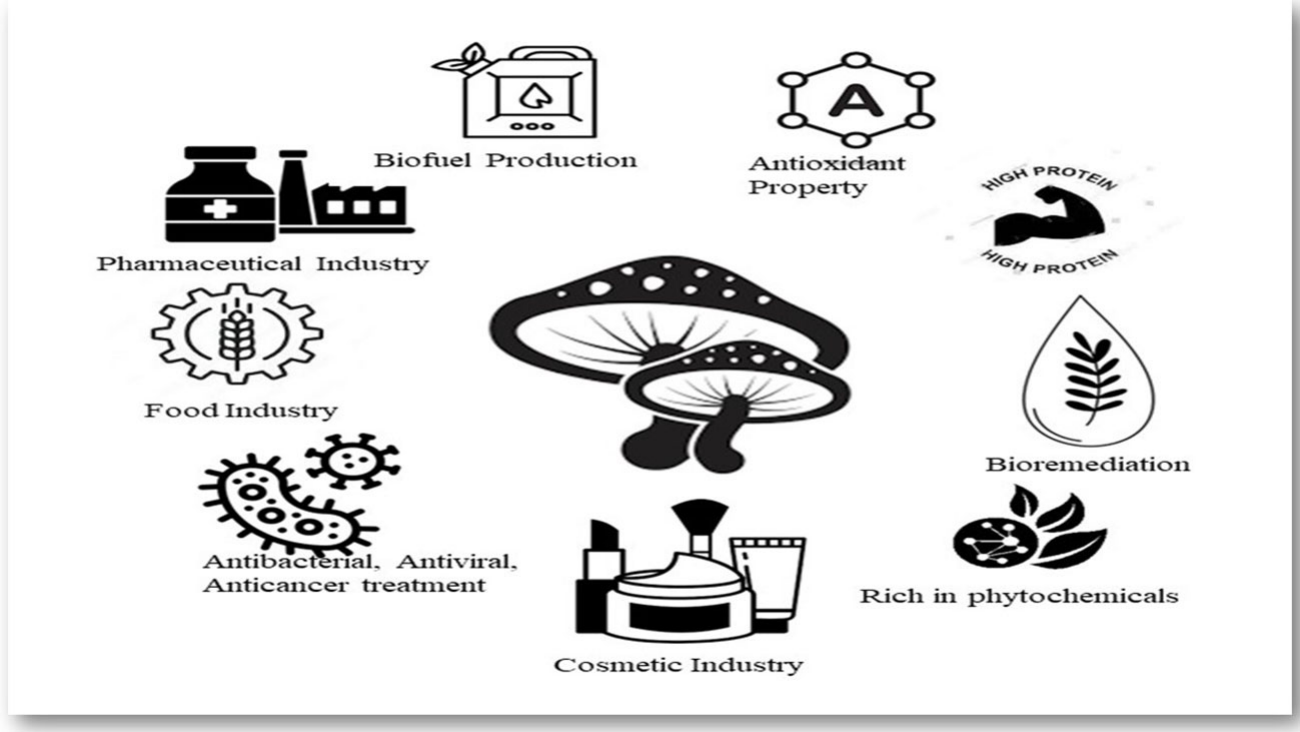
Represents a few applications of mushrooms in different industries.

The number of fungal species on Earth has been estimated to vary from 500,000 to over 5 million, and currently only 20 kinds of mushrooms have been industrially cultivated, while 35 species have been grown commercially (Hawksworth and Lücking, 2017; Bhambri et al., 2022). As a result, it is more important than ever to separate and recognize the unique bioactive substances that higher fungi produce and to identify the exact chemical that will have the desired medicinal effect. Beyond their nutritional value, mushroom metabolites have been shown to exhibit a plethora of bioactive properties such as immunomodulatory action, prebiotic activity, antibacterial (Selamoglu et al., 2020), antiviral activity (Chuang et al., 2020), anti-aging-properties, anti-hypoglycemic activities (Afiati et al., 2019), neuroprotective (Sevindik et al., 2021), antioxidant (Sevindik et al., 2017, 2018), anti-inflammatory (Bucić-Kojić et al., 2020), anticancer (Lowenthal et al., 2023), antimicrobial (Kothiyal and Singh, 2022), and antiparasitic activity (e.g., against *Leishmania* sp.) (Leliebre-Lara et al., 2016), anti-oxidant, as well as anti-*Toxoplasma gondii* properties (Sharma et al., 2023) etc. Mushrooms can also be used as pollution indicators. A study by Akgul et al. (2017) assessed the oxidative stress indices (OSI) in *Auricularia auricula* and *Trametes versicolor* that were collected from Köyceğiz-Muğla, Turkey. Their findings revealed high OSI values in both mushroom species. It was determined that eating mushrooms harvested in certain areas may result in health issues for individuals.

The medicinal mushroom that has been the subject of extensive research in the last 10 years is the fungus *Trametes*, widely distributed throughout Ireland and Britain (Grand and Vernia, 2002; Bhambri et al., 2022). It can be found throughout the European continent, ranging from northern Scandinavia to the Mediterranean region. Asia and North America are also home to this wood-rooting fungus (Habtemariam, 2020). Despite being the most extensively researched and powerful medicinal mushroom, *Trametes* sp. is not yet standardized in terms of cultivation and extraction of maximum β-glucan production, although it has well-known antiviral (Shi et al., 2022) and anti-cancerous properties.

The species is a member of the family *Polyporaceae* and phylum *Basidiomycota* (PUIA et al., 2018), classified in **Table 1**. *Trametes* sp. has been used for millennia in traditional medicine and is now included in cancer treatments (Yang et al., 1992; Piotrowski et al., 2015). The most well-studied biological activity carriers are polysaccharopeptides (PSP) derived from *Trametes versicolor*, which has been renowned as medicine in China and Japan (Cui and Chisti, 2003). PSP, a polysaccharide peptide, and polysaccharide krestin (PSK), a protein-bound polysaccharide, are the two immunologically active components (Habtemariam, 2020). Interestingly, it has been demonstrated that oral administration of PSP and PSK has an anti-cancer effect *in vivo* (Awadasseid et al., 2017).

**Table 1:** Classification of mushroom *Trametes* sp. data obtained from the CABI Bioscience Database of Fungal Names (Kirk, 2021).

Kingdom	Fungi
Phylum	*Basidiomycota*
Class	*Agaricomycetes *
Order	*Polyporales *
Family	*Polyporaceae *
Genus	*Trametes*

Owing to the existence of secondary metabolites, mushrooms possess remarkable biological properties, and as a result, they are used in various industries. **Figure 1** lists industries where mushrooms are employed.

Secondary metabolites isolated from *Trametes* sp., such as phenolic compounds, polysaccharides, terpenoids, and other compounds, are responsible for approximately 130 biological activities, which include immunomodulatory, hepatic protective, antioxidant, antimicrobial, antiviral, and anti-aging properties (Wasser, 2014), among which glycoconjugates are usually considered the main bioactive component (Wang et al., 2019).

Further, *C. versicolor* polysaccharopeptides have been associated with various physiological effects, such as immunopotentiation by triggering the release of interleukin-6, interferons, immunoglobulin-G, macrophages, and T-lymphocytes; antagonizing the immune-suppressive properties of chemotherapy, radiotherapy, and blood transfusion; antagonizing immune system suppression caused by tumors; and preventing the development of various cancers by inducing the production of superoxide dismutase (Dou et al., 2019). Additionally, the State Food and Drug Administration of China (SFDA) has approved 12 different types of *Coriolus versicolor*-based medications for clinical usage thus far (Chang et al., 2017). **Table 2** lists a few medicines and health products derived from *Coriolus versicolor.*

**Table 2:** Some of the *Coriolus versicolor-*based medicine and health products that the China State Food and Drug Administration (SFDA) has authorized for use in clinical and commercial environments.

Trametes sp.	Product	Reference
*Coriolus versicolor* LH1	*Coriolus versicolor* Gantai Granules	Barreras-Urbina et al., 2023
*Coriolus versicolor* COV-1 strain	Gansukang Capsules	Chang et al., 2017
*Coriolus versicolor* COV-1 strain	Polystictus Glycopeptide Capsules	Barreras-Urbina et al., 2023
*Coriolus versicolor* COV-1 strain	Posaverptidum	Barreras-Urbina et al., 2023
*Coriolus versicolor* COV-1 strain	Shen Qi *Coriolus versicolor* Granules	Barreras-Urbina et al., 2023
*Coriolus versicolor* COV-1 strain	*Yikang Versicolor* Capsules	Barreras-Urbina et al., 2023

However, the majority of research has concentrated on the antioxidant properties of a particular wild mushroom species, while some studies have also looked at the antioxidant, immunostimulatory, and anticancer properties of mushrooms. As a consequence, there is now a large research void in the detailed analysis and comparative discussion of β-glucans across a wide variety of commercially cultivated mushrooms.

Therefore, our study aimed to investigate the composition of mycelium biomass and mushroom (broth) extract obtained by the collection of the medicinal mushroom *Trametes* sp. in the Himalayan region, especially in Uttarakhand, and their submerged cultivation, which had previously been studied for its various applications in the pharmaceutical industry. The evaluation of the antioxidant activity of biomass and mushroom extracts, together with the screening of the phytochemical and polysaccharide content of *Trametes* sp. in ethanol extracts using the high-performance liquid chromatography (HPLC) method and a PC-based UV-Vis spectrophotometer, were studied. In addition, we examined the amount of β-glucan in the mycelium that was acquired from submerged cultivation and verified it using the mushroom and yeast β-glucan assay kit (Megazyme Int., Dublin, Ireland).

## 2 Materials and methods

### 2.1 Sample collection

The macrofungal strain fruiting body was collected from the Neelkantha forest, Rishikesh, Uttarakhand, situated at an altitude of 6,596 meters, latitude 30°43′48′′N, and longitude 79°24′20′′E.

### 2.2 Establishment of tissue culture and submerged cultivation of *Trametes* sp.

Fruiting bodies were cleaned, and the inner mass of the mushroom was removed with a sterilized knife and cultivated for 14 days at 20°C in a suitable potato dextrose agar (PDA) medium. A pure culture was obtained and sustained after additional tissue culture was established and multiple subcultures were carried out to purify the culture. After generating further sub-cultures from the pure culture medium, the biomass was grown in a liquid medium (potato dextrose broth; PDB) in a 250-mL volumetric flask and incubated for 14 days at 20°C in the dark. This step was done in batches. Following that, biomass was filtered and dried at 50°C for 2 h. Using a pestle and mortar, the dried biomass was ground into a fine powder. The resulting powder was then used and prepared for analysis. The pure culture was stored in a suitable potato dextrose agar medium at 4°C and was subcultured every 30 days onto the fresh medium.

### 2.3 Amplification of the LSU F/R-rDNA fragment and phylogenetic analysis

Genomic DNA was extracted from cultured samples by a simple lysis protocol, as described in Karn et al. (2011). In total, 125 ηg of extracted DNA is used for amplification, along with 10 pM of each primer. LSU F/R-rDNA fragment was amplified using the ABI 3130 Genetic Analyzer with the following set of primers: forward primer: 5’-TCCTGAGGGAAACTTCG-3’ and reverse primer: 5’-ACCCGCTGAACTTAAGC-3’. The reaction mixture of PCR contained 10x Taq DNA polymerase Assay Buffer, 2.5 mM each dNTP, 3.2 mM MgCl_2_, and 3 U/ml Taq DNA Polymerase in a final volume of 30 ml sterile MQ water. The PCR was performed with an initial denaturation at 94°C for 3 min, followed by 30 cycles of 94°C for 1 min, 50°C for 1 min, and 72°C for 2 min, and a final extension of 72°C for 7 min. The amplification product was gel-purified using ABI 3130xl gel sequencing. The PCR product was sequenced bi-directionally. The sequence data were aligned and compared against the available DNA sequences from type strains in GenBank (http://www.ncbi.nlm.nih.gov/) using the BLAST sequence match tool and analyzed to identify the mushroom and its closest neighbors joining by making a phylogenetic tree using the software Phylogenetic Tree Builder (Bruno et al., 2000).

### 2.4 Preparation of mycelial biomass extract and broth extract

Ethanol (80%, v/v) was used as an extracting solvent for obtaining MBE and BE. The dry biomass (10g) was ground and mixed with 100 ml of 80% ethanol, then left in a laboratory shaker at 25°C and 140 rpm for 24 h. After that, extracts were separated from the mycelium biomass by centrifugation. The process was then carried out once more after the solid had been redissolved in ethanol. Next, Whatman paper No. 4 was used to combine and filter the liquid. In a vacuum evaporator, the filtrate was concentrated, and the thick concentrated extract was collected and used for phytochemical and polysaccharide quantification research.

### 2.5 Phytochemical analysis

#### 2.5.1 Total protein analysis

The total protein content in MBE and BE was determined using Lowry’s assay. An aliquot of 1 mL of extract is treated with 5 mL of Reagent A and kept in the dark for 10 min. Then the mixture was loaded with 0.5 mL of Folin-Ciocalteu reagent and left in a dark place for 30 min. After incubation, the absorbance was measured at 630nm. Protein concentration (mg/mL) was determined using the Bovine Serum Albumin (BSA) standard curve in the extracted samples.

Reagent A is made by combining 99 mL of reagent B (2% Na_2_CO_3_ and 0.1N NaOH), 0.5 mL of reagent C (2% of copper sulfate pentahydrate), and 0.5 mL of reagent D (2% of potassium sodium tartrate).

#### 2.5.2 Total carbohydrate analysis

The total carbohydrate content of extracted samples was determined using the Anthrone technique, with glucose (10–80 mg/L) used as the standard. For the test, 1 mL of extract is treated with 5 mL of the anthrone reagent (dissolve 2g of anthrone in 1000 mL of concentrated sulfuric acid) and incubated at 100°C for 10 min. After incubation, the absorbance was measured at 620nm. Carbohydrate concentration (mg/mL) was determined using the glucose standard curve in the extracted samples.

#### 2.5.3 Total flavonoid analysis

The total flavonoid content (TFC) of fungal MBE and BE extracts was evaluated according to the method described by Wandati et al. (2013). An aliquot of 1 mL of the sample was added to 0.3 mL of 5% NaNO_2_, and 4 mL of distilled water was kept for 5 min. Then the mixture was loaded with 0.3 mL of 10% AlCl_3_ and allowed to sit for 6 min. Finally, 2 mL of 1 M NaOH was added to these tubes, followed by 10 ml of distilled water. The absorbance was measured at 415nm. Quercetin was used as a standard in a concentration range of 20–100 µg/mL, and the result was expressed as µg QE/g DW.

#### 2.5.4 Total phenol analysis

The total phenol content (TPC) of the fungal MBE and BE extracts was analyzed using the Folin-Ciocalteu method of Wandati et al. (2013), with some modifications. Each sample (1 mL) was mixed with 0.5 mL of 10% (v/v) Folin-Ciocalteu phenol reagent and 2 mL of 700 mM Na_2_CO_3_ in distilled H_2_O. The mixture was vortexed well and left for 30 min. After incubation, the absorbance was measured at 765nm. The TPC in the extract was expressed as μg gallic acid equivalent (GAE) per g dry weight (μg GAE/g DW).

#### 2.5.5 Total saponin analysis

The total amount of saponin in fungal MBE and BE extracts was analyzed as described by Le et al. (2018). An aliquot of 1 mL of the sample was added to 1 mL of 8% vanillin and 2.5 mL of 72% sulfuric acid. The mixture was incubated for 15 min at 60°C. After incubation, the absorbance was measured at 560nm. Aescin was used as a standard in a range of 20–100 µg/mL, and the result was expressed as mg Aescin/g DW.

#### 2.5.6 Total anthraquinone analysis

The total anthraquinone content of fungal MBE and BE extracts was evaluated according to the method described by Sakulpanich and Gritsanapan (2008), with some modifications. In 1 mL of sample, 10 mL of distilled water was added. Then, 20 mL of 10.5% ferric chloride was added, and the mixture was mixed and refluxed for 10 min. Add 1 mL of sulfuric acid and reflux for another 10 min. Transfer the final content into a separating funnel, and extraction was done three times with 25 mL of ether. After that, the ether layer was evaporated to dryness to collect the residues. The residues were dissolved in 1 ml of 0.5% Mg(CH_3_COO)_2_ in CH_3_OH, and absorbance was measured at 515 nm. Rhein was used as a standard in a range of 20–100 µg/ml, and the results were expressed as µg Rhein/g DW.

#### 2.5.7 Total alkaloid analysis

The total alkaloid content in macrofungal MBE and BE extracts was determined as described by Nantongo et al. (2018). A total of 50 mL of 10% acetic acid in ethanol was added to 100 mg of the sample; the mixture was covered and incubated for 4 h. After that, the extract was concentrated in a water bath to one-quarter of the original volume, followed by the addition of five drops of concentrated NH_4_OH to precipitate alkaloids. The precipitate was allowed to rest for 3 h to sediment before being rinsed with 10 mL of 0.1M NH_4_OH and filtered. The residue on the paper was dried at 60°C for 30 min and then reweighed. Mathematically, the % of the alkaloid is calculated as mentioned in Equation 1:


(1) Alkaloid percentage=weight of residue/weight of sample∗100

#### 2.5.8 Terpenoids analysis

The terpenoid content in macrofungal MBE and BE extracts was determined as described in Malik (2017). A total of 9 mL of ethanol was added to 100 mg of extract and incubated in the mixture for 24 h. Using the separating funnel, the extract was filtered with 10 mL of petroleum ether. A pre-weighed glass vial was used to collect the ether extract. At room temperature, the solvent was allowed to completely evaporate and then reweighed. The % terpenoids content was measured using the formula given in Equation 2:


(2) Terpenoids Percentage= weight of residue/weight of sample ∗100

#### 2.5.9 Total tannin analysis

The total tannin content in macrofungal MBE and BE extracts was determined using the Folin-Denis reagent. 10 mL of distilled water was added to 1mg of extract, gently boiled for 1 h in a water bath, and filtered using Whatman filter paper in a 10 mL volumetric flask. To this content, 5 mL of Folin-Denis reagent and 10 mL of saturated Na_2_CO_3_ solution were added and incubated at 25°C in a water bath for 30 min. After incubation, the absorbance was measured at 700 nm. The amount of tannin in the extracts was calculated using the equation developed on the standard curve of tannic acid.

#### 2.5.10 Sterols analysis

The sterol content in macrofungal MBE and BE extracts was determined using the Liebermann-Burchard reagent, and cholesterol was used as the standard concentration range of 0.2–1 mg/mL. In 1 ml of acetic acid, 1 mg of the extract was dissolved. Then, it was treated with 5 mL of Liebermann-Burchard reagent and incubated at 37°C in a water bath for 10 min. After incubation, the absorbance was measured at 650 nm.

#### 2.5.11 Antioxidant property of the 2,2-diphenyl-1-picrylhydrazyl assay

The ability of the MBE and BE extracts to donate electrons and scavenge DPPH radicals was determined by the slightly modified method of Anjana et al. (2016). A freshly prepared 1 mM ethanol solution of DPPH was mixed with the mycelium biomass and mushroom extract at a ratio of 1:0.5 (v/v). The mixture was vortexed properly and incubated in the dark for 10 min. The light absorption was measured at 517 nm. The % scavenging value at the variable concentration was used to plot a regression curve, and the equation generated on the curve was used to estimate the IC_50_ value for both extract samples. The Equation 3 is used for % scavenging calculation:


(3) Scavenging percentage = absorbance of control – absorbance of sample * 100

#### 2.5.12 β-glucan analysis

The amount of β-glucan in fungal MBE and BE was measured based on the enzymatic release of glucose, as described in Toledo et al. (2013). This procedure calls for a standard reactive glucose solution with a concentration of 100 mg dL^-1^, an enzyme reactive mixture with glucose oxidase and peroxidase, color reagent 1 (4-aminophenazone, 25 mM, 920 mM Tris), and color reagent 2 (55 mM phenol). The working reagent was prepared in a 250-mL volumetric flask using 225 mL of distilled water, 12.5 mL of color reagents 1 and 2, and 0.75 mL of enzyme reactive. In total, 20 mL of each sample extract was added to 2 mL of working reagent in the reaction mixture and incubated at 37°C for 15 min. After that, the absorbance at 505 nm was determined using a spectrophotometer. Using 2 mL of the working reagent and 20 mL of standard reactive glucose, the reaction was carried out under the same conditions as above. The results were examined using Equation 4 below:


(4) β–glucan (mg/dL)=A * f * 0.9

Where A denotes the absorbance of the sample at 505 nm, which is equal to 100 mg/dL of normal glucose, and 0.9 denotes the proportion of free glucose conversion that was established for anhydrous glucose, which occurs in β-glucan.

#### 2.5.13 Glucan content analysis using the Megazyme kit

The contents of the total α- and β-glucans were determined in the MBE and BE using the mushroom and yeast β-glucan assay kit (Megazyme Int., Dublin, Ireland), following the instructions of the manufacturer. Briefly, to estimate the total glucan content in the samples, 2 mL of ice-cold 12M sulfuric acid was used for the hydrolysis of the polysaccharides in the samples at 100°C for 2 h. After neutralization, hydrolysis proceeded to glucose using a mixture of exo-β-(1,3)-D-glucanase + β-glucosidase in sodium acetate buffer (pH 4.5) at 40°C for 1 h. For α-glucan content estimation, enzymatic hydrolysis with amyloglucosidase and invertase was conducted. After that, a glucose oxidase/peroxidase reagent was added to estimate the total glucan and α-glucan content at 510nm. The β-glucan content was calculated by subtracting the α-glucan from the total glucan content. All values of total, α-, and β-glucans in biomass and mushrooms were expressed in g/100g of DW.

**Determination of β-glucan**: The β-glucan content was determined by subtracting the α glucan content from the total glucan content.

#### 2.5.14 Quantification of phytochemical content by HPLC

The identification and quantification of phytochemicals from mycelium biomass and mushroom extracts were performed using the high-performance liquid chromatography (PDA) technique. Chromatography was performed on a 250mm x 4.5mm RP C-18 column. Preliminary experiments, in which various ratios of methanol and water were examined, helped to optimize the condition. Quercetin, sesquiterpene glycosides, ergosterol, and RidentinB were the standards employed for the identification and quantification of total polyphenols, terpenoids, sterols, and sesquiterpenes, respectively. The flow rate, column temperature, and detector wavelength were all adjusted to 1 mL/min, 25°C, and 210 to 370 nm, respectively.

The formula used for calculation is mentioned in Equation 5:


(5) $$\begin{array}{l} \text{The concentration of compound }\left(\text{mg}/\text{mL}\right)=\left(\text{sample absorbance}/\text{mean absorbance of standard}\right)\\ \;*\;\left(\text{standard dilution}/\text{sample dilution}\right)\;*\;\left(\text{standard potency}/100\right)\;*\;100. \end{array}$$

### 2.6 Statistical evaluation

All the experiments were conducted in triplicate, and the values were expressed as mean ± SD. Statistical significance was detected by the Statistical Package for the Social Sciences (SPSS); a value of p<0.05 indicated a statistical difference.

## 3 Result

**Figure 2:**
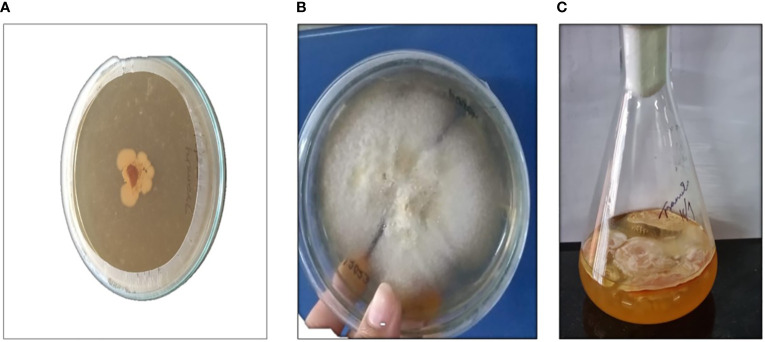
Represents *Trametes* sp. **(A)** Mycelium grown on potato dextrose agar medium (PDA); **(B)** After 14 days of incubation; **(C)** Mycelium grown on potato dextrose broth medium (PDB).

In the present study, the submerged cultivation of *Trametes* sp. was conducted in a liquid (PDB) and solid medium (PDA). *Trametes* sp. grew on a PDA plate covered with mycelium, and the cultivation lasted 14 days at 20°C. In the broth medium, the grown culture formed a layer of mycelium on top of it, and relatively high quantities of mycelium biomass were produced, as represented in **Figure 2C**. *Trametes* sp. resembles the tail of a strutting turkey, having arched bands of brown, tan, and white color, as depicted in **Figures 2A** and **B**. According to studies, submerged cultivation offers a substitute method for consistently obtaining macrofungal biomass that produces essential metabolites, as well as a technique to lower downstream processing costs, lower the risk of contamination, and ensure sustainability (Tang et al., 2007; Lu et al., 2020; Bakratsas et al., 2021). The production of biomass was highly dependent on the strain, growth methods, and recovery procedures. We reported a high amount of biomass, 96.96 g/L, and after drying the biomass at 50°C for 2 h, we recovered 3 g DW/L. In a work by Angelova et al. (2022), they used a lyophilized approach to achieve 10.22 ± 0.28 g DW/L of mycelium biomass. However, there are still many obstacles in the way of its cultivation and extraction of phytochemicals and polysaccharides, especially β-glucan from *Trametes* sp. So far, there is very little comparative study that includes analysis of most of the valuable phytochemicals and polysaccharides, mainly β-glucan in *Trametes* sp. from both MBE and BE.

### 3.1 Molecular identification

**Figure 3:**
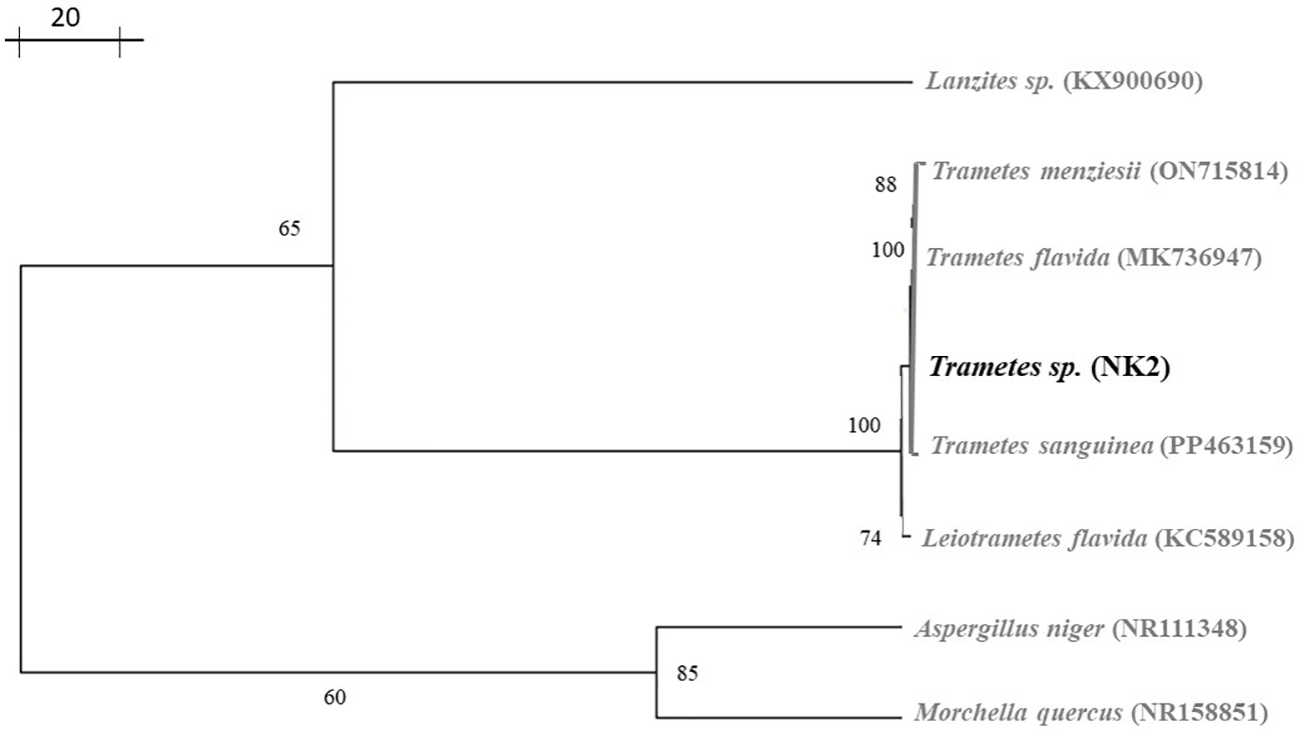
Shows a phylogenetic tree based on the LSU F/R-rDNA fragment (NK-2; highlighted in bold).

Wild mushrooms have highly variable morphological features; identifying them solely based on physical characteristics may be deceptive. According to Hussein et al. (2014); Khatun et al. (2017); Raja et al. (2017), and Ao et al. (2020), combining morphological and molecular data was the most effective way to advance the field of mushroom identification research. Here, we performed a partial sequence of the isolates, analyzed by BLAST search. The BLAST analysis of this sequence showed 100% sequence similarity with the *Trametes flavida* voucher OAB0196 large subunit ribosomal RNA gene (**Table 3**). A phylogenetic tree was constructed based on LSU F/R-rDNA fragment neighbor-joining using Phylogenetic Tree Builder software. The phylogenetic analysis revealed that NK-2 (mushroom sample) was found to be the closest homolog to the *Trametes flavida* voucher OAB0196 large subunit ribosomal RNA gene with sequence ID: MK736947. The next closest homolog was found to be the *Trametes flavida* voucher OAB0047 large subunit ribosomal RNA gene with sequence ID: MK736946, hence designated as *Trametes* sp. NK-2 (**Figure 3**).

**Table 3:** The BLAST sequence analysis.

Serial No.	Organism name	Accession No.	% Match
**1**	*Trametesflavida* voucher OAB0196 large subunit ribosomal RNA gene	MK736947.1	100.00%
**2**	*Trametesflavida* voucher OAB0047 large subunit ribosomal RNA gene	MK736946.1	100.00%
**3**	*Daedaleaflavida* strain CBS 158.35 large subunit ribosomal RNA gene	MH867126.1	100.00%
**4**	*Lenzites*sp. voucher Dai 1310328S ribosomal RNA gene	KX900690.1	100.00%
**5**	*Leiotrametesflavida* strain DMC813 28S ribosomal RNA gene	KC589158.1	100.00%
**6**	*Leiotrametesflavida* strain DMC812 28S ribosomal RNA gene	KC589157.1	100.00%
**7**	*Leiotrametes flavida* strain DMC811 28S ribosomal RNA gene	KC589156.1	100.00%
**8**	*Trametes cf. cubensis* Dai 11582 25S large subunit ribosomal RNA gene	KC848408.1	100.00%
**9**	*Trametes cf. cubensis* Dai 7861 25S large subunit ribosomal RNA gene	KC848407.1	100.00%
**10**	*Daedaleopsisflavida* strain 5A 5.8S ribosomal RNA gene.; internal transcribed spacer 2, complete sequence; and 28S ribosomal RNA gene	JF712849.1	99.74%

### 3.2 Characterization of MBE and BE

It is important to note that the chemical composition of macrofungal biomass is strain-specific and strongly dependent on the environment and cultivation technique (Bains et al., 2021). These days, numerous techniques can help in the extraction process, such as methanol extraction (Sezer et al., 2017), enzyme assistance, microwave assistance, ultrasonic assistance, ethanol extraction (Bae et al., 2015; Angelova et al., 2022), and hot water extraction (Park and Hong, 2014). In this study, we used an 80% ethanol extraction method to extract pure phytochemicals and polysaccharides from MBE and BE. Further, this method is to be optimized and compared with the classical ethanol precipitation procedures.

### 3.3 Protein and carbohydrate content

The total protein content found in the BE and ME extracts of *Trametes* sp. in our study was 0.0153 and 0.1088 mg/mL, respectively, as represented in **Table 4**. However, in the report, crude protein content for basidiomycetes varies between 10% and 49% (Bakratsas et al., 2021; Angelova et al., 2022). In our study, we found 10% protein content in biomass, which falls below the reported range. Since cultivation techniques and substrate composition strongly affect the chemical composition of mushrooms, including protein content, research is constantly sought for new sources of proteins to meet the increasing protein demand of a continuously growing world population. Mushrooms can serve as an important source of protein because they can provide all essential amino acid requirements.

**Table 4:** The amount of total protein and carbohydrates in BE and MBE extracts.

Serial No.	Sample	Total protein (mg/mL)	Total carbohydrate (mg/mL)
**01**	BE	0.0153	0.0257
**02**	MBE	0.1088	0.0184

On the other hand, polysaccharides were only partially soluble since the total carbohydrate content in BE and MBE extracts reported in our study was 0.0257 and 0.0184 mg/mL, respectively, as depicted in **Table 4**. Hence, the polysaccharide solubility could be negatively affected by precipitation or drying methods, and its decrease was probably due to conformational changes.

The phytochemical compositions of the MBE and BE extracts were used to be analyzed by the systronic PC-based UV-Vis spectrophotometer 220.

The results of flavonoid in BE and MBE extracts were 11.16 µg/mL and 9.50 µg/mL, respectively, and the results of TPC in BE and MBE extracts were 12.7 µg/mL and 12.4 µg/mL, respectively, as represented in **Table 5**. Our results show high TFC and TPC content in 80% ethanol extraction compared to the result reported by Angelova et al. (2022) in methanol and ethanol extraction for both TPC and TFC; however, their water extraction proved to be a good choice in *T. versicolor* biomass treatment.

**Table 5:** The content of total flavonoids, phenolic content, saponins, and anthraquinones in BE and MBE extracts.

Serial No.	Sample	Total flavonoid content (µg/mL)	Total phenolic content (µg/mL)	Total saponin content (µg/mL)	Total anthraquinone content (µg/mL)
01	BE	11.16	12.7	8.6	13.5
02	MBE	9.50	12.4	70.6	14.5

Other phytochemicals, such as saponin and anthraquinones, obtained the highest content among all phytochemicals in MBE compared to BE. Saponin was found to be the dominant phytochemical in biomass extract. The results of saponin in BE and MBE extracts were 8.60 µg/mL and 70.6 µg/mL, respectively, against the standard Aescin concentration range of 20–100 µg/mL. For anthraquinones, the standard uses a Rhein concentration range of 20–100 mg/mL. After saponin, anthraquinone has the second-highest content in MBE at 14.5 and 13.5 µg/mL in BE extract, as represented in **Table 5**. The result also suggests that the condition provided in submerged cultivation for biomass extraction was good; therefore, we obtained a high amount of saponin and anthraquinone content in biomass extract rather than mushroom extract. Similarly, Ogidi et al. (2021) carried out the anti-microbial efficacy of wild edible mushrooms, where they found saponin content was 0.15 ± 0.01%, suggesting the usefulness of the mushroom as a potential antimicrobial, anticarcinogenic, antimalarial, antiulcer, and hepaticidal effect. On the other hand, phytochemical studies by Doris (2018) on *Trametes* sp. suggest that they did not find anthraquinone content in ethanol extraction.

The percentage of alkaloid and terpenoid content was calculated using Equations 1 and 2, respectively. The results of alkaloids were 1.21% and 1.53% in BE and MBE extracts, respectively, and the results of terpenoids were 3.65% and 4.21% in BE and MBE extracts, as mentioned in **Table 6**. However, Doris (2018) phytochemical studies on *Trametes* sp. collected from Ondo, Akure, and Ipele districts in Ondo State in southwest Nigeria, using extractions from three solvents—ethanol, ethyl acetate, and hexane—determined the qualitative and quantitative analysis of nine secondary metabolites (alkaloids, tannins, saponins, flavonoids, terpenoid, steroid, and phlabotannin). All of the secondary metabolites examined were present in all of the mushrooms investigated, except for the alkaloids, phlabotannin, and anthraquinone.

**Table 6:** The content of alkaloids, terpenoids, tannins, and sterols in BE and MBE extracts.

Serial No.	Sample	Alkaloid (%)	Terpenoid (%)	Tanin (mg/mL)	Sterol (mg/mL)
01	BE	1.21%	3.65%	0.0778	1.314
02	MBE	1.53%	4.21%	0.0882	1.592

Similarly, the results of tannin in BE and MBE extracts are 0.0778 and 0.0882 mg/mL, respectively, as represented in **Table 6**. However, Chaudhary et al. (2023) reported six wild mushrooms: *Scleroderma citrinum, Suilluspunctatipes, Coriolushirsutus, Russulasanguinea,Heterobasidionannosum*, and *Cavimalum indicum*, of which *S. punctatipes* had the maximum amount of tannin concentration (180 mg GAE/g).

The results of the sterol content in BE and MBE were 1.314 mg/mL and 1.592 mg/mL, respectively, as represented in **Table 6**.

In the present study, the antioxidant activity was assessed by the DPPH method. The IC_50_ value was used to measure antioxidant activity, and the IC_50_ value drops as antioxidant activity rises. As shown in **Tables 7** and **8**, MBE exhibited the highest antioxidant activity since its IC_50_ value is 405.042 µg/mL, while BE’s IC_50_ value is 376.032 µg/mL, suggesting the presence of a high content of TPC, TFC, saponin, and anthraquinone in biomass extract.

**Table 7:** The % scavenging potential of BE at different concentrations (DPPH).

Extract concentrations (µg/mL)	Sample Abs at 517nm	% Scavenging	IC50 value
100	0.263	18.069	376.032 µg/mL
200	0.219	31.776	
300	0.182	43.302	
400	0.155	51.713	
500	0.119	62.928	

**Table 8:** The % scavenging potential of MBE at different concentrations (DPPH).

Extract concentrations (µg/mL)	Sample Abs at 517nm	% Scavenging	IC50 value
100	0.273	14.953	405.042 µg/mL
200	0.230	28.349	
300	0.193	39.875c	
400	0.162	49.533	
500	0.131	59.190	

Sun et al. (2014) discovered six polysaccharide fractions of *Coriolus versicolor* (CVPS) among CVPS with low molecular weight, high protein content, and greater uronic acid content that showed a high radical scavenging effect, with CVPS-3 having the highest antioxidant activity and CVPS-3 exhibiting higher DPPH radical scavenging activity (64.9% at 0.8 mg/mL), respectively. The percentage of scavenging activity in BE and MBE extracts was calculated as mentioned in Equation 3. Compared with our result, *Trametes* sp. BE extract exhibits higher DPPH radical scavenging activity (62.9% at 0.5 mg/mL) than MBE (59.19% at 0.5 mg/mL).

In the current study, the content of β-glucan was calculated using Equation 4 in both MBE and BE obtained by submerged cultivation of *Trametes* sp. The MBE showed a higher β-glucan content of 1.713 mg/mL than the BE, which is 1.671 mg/mL, as reported in **Table 9**. Depending on the species, habitat, maturity, and cultivation techniques, the percentage of fiber found in the fruiting body or mycelium of a mushroom can range from 3.1% to 46.5% dry matter (DM) (Latgé, 2007; Kumari, 2020).

**Table 9:** The concentration of β-glucan content in BE and MBE extracts.

Serial No.	Sample	β-glucan content (mg/mL)
01	BE	1.671
02	MBE	1.713

High-pressure liquid chromatography examination.

Further, to confirm the yield of β-glucan in *Trametes* sp. determined using the Mushroom and Yeast β-glucan assay kit (Megazyme Int., Dublin, Ireland), we obtained 42.5% in MBE. In our analysis, 300 mg of MBE contains total glucans 45.2025 (%w/w), α-glucan 2.6406 (%w/w), and β-glucan 42.5 (%w/w). However, in BE, the yield of β-glucan is comparatively low, 5.94%, which suggests that biomass extract could provide a good alternating source to obtain maximum pure β-glucan from *Trametes* sp.

**Figure 4:**
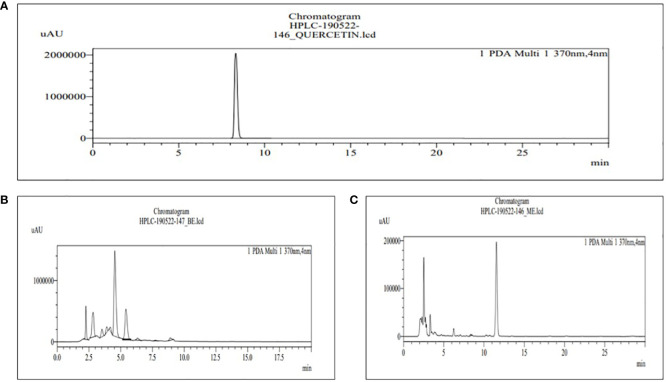
HPLC chromatograms of the standard quercetin **(A)** and the extract of MBE and BE **(B)** and **(C)**, respectively. For the identification and quantification of phytochemical total polyphenols, methanol and 0.4% phosphoric acid (47:53) are used in the mobile phase.

**Figure 5:**
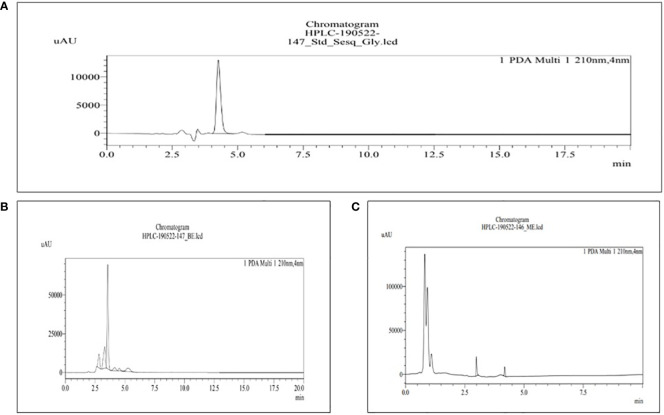
HPLC chromatograms of the standard sesquiterpene glycosides **(A)** and the extract of MBE and BE **(B)** and **(C)**, respectively. For the identification of phytochemical terpenoids, 0.1% formic acid and acetonitrile (60%–40%) are used in the mobile phase.

**Figure 6:**
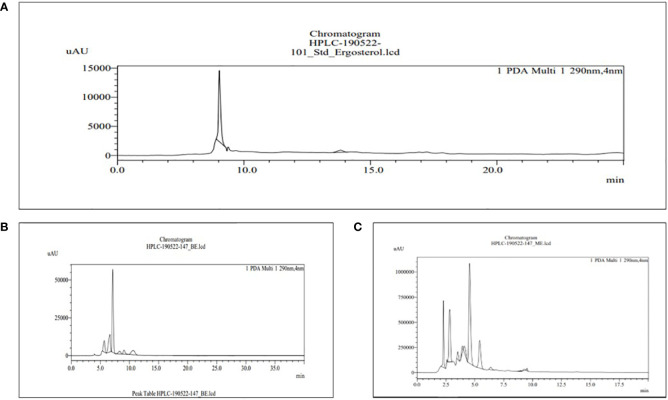
HPLC chromatograms of the standard ergosterol **(A)** and the extract of MBE and BE **(B)** and **(C)**, respectively. For the identification and quantification of phytochemical sterols, methanol and acetonitrile (80:20) are used in the mobile phase.

**Figure 7:**
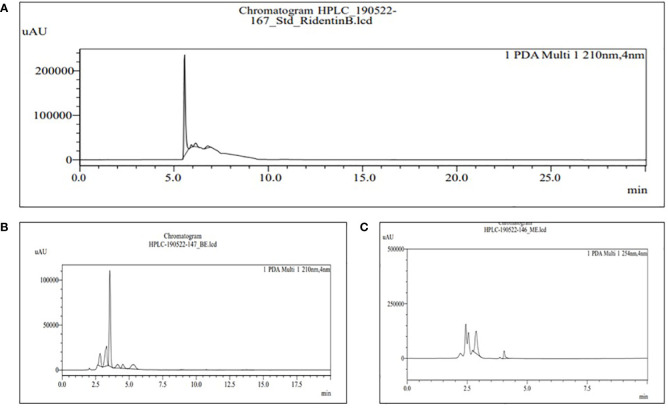
HPLC chromatograms of the standard RidentinB **(A)** and the extract of MBE and BE **(B)** and **(C)**, respectively. For the identification and quantification of phytochemical sesquiterpene, 60% acetonitrile water is used in the mobile phase.

The HPLC was used for the analysis of phytochemical total phenols, terpenoids, sterols, and sesquiterpene in both BE and MBE, represented in **Figures 4**–**7**, respectively, and their concentrations were measured using Equation 5, along with their standard (1 mg/mL). Peaks were detected by comparing them to the retention times of their respective standards. Due to a lack of standards, however, many of the compounds could not be identified. The peak that shows similarity with the standard one has that particular component that we were looking for in the sample; the other peak represents some other compounds that we will investigate in our next studies.

The retention time for the standard quercetin was 8.320, as shown in **Figure 4A**, used for total phenolic content in MBE and BE extracts. Peak 10 with a retention time of 8.396 min represents MBE, as shown in **Figure 4B**, and peak 8 with a retention time of 8.327 min represents BE, as shown in **Figure 4C**. Bristy et al. (2022) also evaluated the phenolic content of three varieties of mushrooms growing in Bangladesh, namely *G. lucidum, G. tropicum*, and *C. indica*, using the HPLC-DAD technique, and their findings show that the locally cultivated mushroom *G. lucidum* has the greatest total phenolic content.

The retention time for the standard sesquiterpene glycosides was 4.256 min, as shown in **Figure 5A**, used for terpenoid content in MBE and BE. Peak 4 with a retention time of 4.259 min represents MBE, shown in **Figure 5B**, and peak 5 with a retention time of 4.205 min represents BE, shown in **Figure 5C**, respectively, as shown in **Figure 3**. According to Peng and Shahidi (2022), screening and characterization of secondary metabolites of Newfoundland chaga mushroom using HPLC-ToF-MS (high-performance liquid chromatography coupled with time-of-flight mass spectra). Their chaga sample contains 108 terpenoids that have been tentatively identified, but only 30 can be recognized. The remaining 78 terpenoids were discovered to be novel.

The retention time for the standard ergosterol was 9.016 min, as shown in **Figure 6A**, used for sterol content in MBE and BE extracts. Peak 6 with a retention time of 9.014 min represents MBE, as shown in **Figure 6B**, and peak 11 with a retention time of 9.015 min represents BE, as shown in **Figure 6C**. Gil-Ramirez et al. (2011) performed an aGC column and observed that, except for *G. lucidum*, all of the analyzed samples contained equal amounts of ergosterol and ergosta-7,22-dienol. The distribution of ergosterol derivatives varied depending on the strain. The selected strain ergosterol concentrations ranged from 0.7 to 5.7 mg/g, with *B. edulis* having the highest levels and *C. cornucopioides* having the lowest.

The retention time for the standard RidentinB used for sesquiterpene content was 5.784 min, as shown in **Figure 7A**; peak 6 represents MBE in **Figure 7B** with a retention time of 5.344 min; and peak 2 represents BE in **Figure 7C** with a retention time of 4.04 min. Further, for the first time, Lee et al. (2020) reported linear sesquiterpene carboxylic acids in *Phallus gluteus*. They used liquid chromatography-mass spectrometry to investigate the methanol extract of *Phallus gluteus* and discovered two novel sesquiterpenes, phallic acid A and B.

Additionally, Gogoi et al. (2019) observed that reverse-phase high-pressure liquid chromatography (RP-HPLC) revealed that mushroom extract had a significant amount of gallic acid, caffeic acid, ascorbic acid, and quercetin and can be considered a good source of natural antioxidants.

## 4 Discussion

Mushrooms have high biological value, help to strengthen the body, flush out toxins, dissipate heat, boost immunity, and uplift the spirit and vitality (Bolla et al., 2010; Feng et al., 2016, 2017; Liu and Zhang, 2018; Yin et al., 2020). *Trametes* sp. possesses a highly bioactive mycelium (Benson et al., 2019). It will be highly beneficial because this fungus has numerous applications in the food (Bakratsas et al., 2021), pharmaceutical (Leliebre-Lara et al., 2015), bioremediation (Han, 2021), cosmetic (Vaithanomsat et al., 2022), and health sectors. Moreover, the macrofungal biomass could be considered more beneficial in detoxification processes and oxidative stress prevention than the pure macrofungal extracts (Valverde et al., 2015). After cultivating various strains of basidiomycetes for 14 days, the most recorded biomass yields varied from 0.1 g/L to 16.0 g/L (Maziero et al., 1999; Jaros et al., 2018). Nevertheless, we reported a high amount of biomass, which is 96.96 g/L, and after drying the biomass, we obtained 3 g of DW/L biomass. A few studies have been conducted on bioactive metabolites from medicinal mushrooms, primarily *Trametes* sp., and those that have attempted to do so have found that the conditions required for the cultivation and extraction of metabolites from mushrooms are costly and hence not economical (Kothiyal and Singh, 2022). As *Trametes* sp. is the most researched and effective medicinal fungus, no established technique exists for cultivating and extracting the maximum quantity of β-glucan from the mushroom.

However, ethanol extraction was discovered to be the most effective method for extracting phytochemicals and polysaccharides from the biomass of mushrooms and their fruiting bodies. Shomali et al. (2019) examined the polyphenolic contents and biological activities of four wild mushrooms and found that the ethanolic extracts of *I. hispidus* had the highest total phenolic and flavonoid contents when compared to other mushroom species. Our results also showed high β-glucan, total phenolic, flavonoid, saponin, and anthraquinone content. The phytochemical screening of *P. ostreatus* by Rahimah et al. (2019) revealed that 70% ethanol extract appeared to contain 6.67 μg/mL total phenolic content, which is almost half of what we found in terms of biomass and mushroom extract. In addition, Nkadimeng et al.’s (Nkadimeng et al., 2020) study on *Psilocybenatalensis* mushroom showed that phytochemical analysis supported the higher beneficial effects of ethanol extracts than water extracts. Furthermore, Rahimah et al. (2019) found that, based on the foam produced, the phytochemical screening revealed that ethanol extract appeared to have the highest concentration of saponin, similar to our result. Similarly, the results of Kothiyal and Singh’s (Kothiyal and Singh, 2022) antimicrobial and phytochemical screening of wild mushrooms that are naturally found in the Garhwal Himalayan region of Uttarakhand, India, indicate that the ethanol extract of *Trametes versicolor* tested positive for terpenoids, steroids, glycosides, flavonoids, carbohydrates, and amino acids; however, the tests for alkaloids, flavonoids, phenolic compounds, carbohydrates (Benedict’s test, Barfoed test), amino acids (Millon’s test, Xanthoproteic), saponins, and inorganic acids were negative. Additionally, the ethanolic extract of *Trametes* sp. exhibits the highest level of total phenolic and flavonoid content when compared to our results.

Recently, research has focused on the many health benefits of dietary fibers with macrofungal origins. β-glucan exhibits potential as a therapy for diabetes and cardiovascular risk. The amount of β-glucan obtained in our work is significantly greater than that of estimates of β-glucan obtained from a variety of mushroom species, such as *Russula alatoreticula* (Khatua et al., 2021), *Polyporus grammocephalus* (Patra et al., 2021), *Pleurotus djamor* (Maity et al., 2019), and *Pleurotus eryngii* (Al-Saffar et al., 2020). There are currently no published reports available from India on the extraction of β-glucan from *Trametes* sp. Moreover, our target audience will be cancer patients, given that the disease has probably affected people for as long as life itself. Al-Saffar et al. (2020) investigated the antioxidant activities of β-glucan that was extracted from *Pleurotus eryngii* using a hot water extraction method; the yield was 7.9%. MCF-7 and HepG2 cell lines were used to test the anticancer activity of β-glucan at several doses. In the cell walls of mushrooms, glucans help to build the structure (Khan et al., 2018; Ruiz-Herrera and Ortiz-Castellanos, 2019). By dry weight, β-glucans make up as much as 50% and α-glucans as much as 10% of the cell walls. The most isolated glucans from mushrooms are β-glucans, which typically have -1,3-glucans with -1,6 branches and have an abundance of important bioactivities (Maity et al., 2017; Yin et al., 2020; Cognigni et al., 2021; Ruthes et al., 2021). β-glucan also exhibited anti-collagenase, anti-elastase, and anti-hyaluronidase activity and can serve as an important component in the cosmetics industry as well (Sangthong et al., 2022).

Concerning mushroom potential medical benefits, our main focus was identifying the maximum phytochemical and polysaccharide contents of biomass extract and mushroom fruiting bodies. The findings we obtained indicated a high concentration of these substances. Yet, there is not a standard extraction procedure for β-glucan derived from mushrooms. In the future, we plan to standardize methods of cultivation and extraction from mushrooms (*Trametes* sp.) to boost the yield of polysaccharides.

## 5 Conclusions

In the present study, *Trametes* sp. submerged cultivation was carried out. The phytochemical and polysaccharide content of mushroom fruiting bodies and biomass were determined, along with the feasibility of using them as healthy food supplements. Our results demonstrate that the medicinal fungus *Trametes* sp. biomass has the highest levels of saponin (70.6 µg/mL), phytochemicals followed by anthraquinone (14.5 µg/mL), total phenolic (12.45 µg/mL), and flavonoid (9.500 µg/mL). Also, the macrofungal biomass extract showed 42% β-glucan content in *Trametes* sp. On the other hand, alkaloid and terpenoid content were the lowest among all the phytochemicals. The findings of this study indicate that phytochemicals and polysaccharides extracted from submerged fungal biomass and fruiting bodies from *Trametes* sp. could represent an intriguing method to obtain minimally processed natural dietary supplements with significant health benefits. The findings of this study suggest that, despite the existence of secondary metabolites that typically have antioxidant action in *Trametes* sp. mushrooms, such as flavonoids, terpenoids, steroids, tannins, saponins, sterols, and total phenol, more research on mushrooms with diverse substrates and variables is needed to boost their phytochemical, polysaccharide, and antioxidant activity.

## Data availability statement

The original contributions presented in the study are included in the article/supplementary material, further inquiries can be directed to the corresponding author/s.

## Author contributions

MS: Writing – review & editing, Resources, Funding acquisition. MK: Writing – review & editing, Data curation, Writing – original draft. SK: Writing – review & editing, Conceptualization, Formal analysis, Resources, Supervision, Validation, Project administration. AB: Writing – review & editing. VM: Writing – review & editing, Funding acquisition, Investigation. SM: Writing – review & editing, Funding acquisition, Investigation.
